# First-line nivolumab plus ipilimumab with or without chemotherapy for Japanese patients with non-small cell lung cancer: LIGHT-NING study

**DOI:** 10.1093/jjco/hyad195

**Published:** 2024-01-25

**Authors:** Hisao Imai, Takashi Kijima, Koichi Azuma, Kazuma Kishi, Haruhiro Saito, Teppei Yamaguchi, Junko Tanizaki, Yasuto Yoneshima, Kohei Fujita, Satoshi Watanabe, Satoru Kitazono, Tatsuro Fukuhara, Osamu Hataji, Yukihiro Toi, Hideaki Mizutani, Yusuke Hamakawa, Makoto Maemondo, Tomoyuki Ohsugi, Keisuke Suzuki, Hidehito Horinouchi, Yuichiro Ohe

**Affiliations:** Department of Respiratory Medicine, Comprehensive Cancer Center, International Medical Center, Saitama Medical University, Saitama, Japan; Department of Respiratory Medicine and Hematology, Hyogo Medical University, School of Medicine, Nishinomiya, Hyogo, Japan; Division of Respirology, Neurology and Rheumatology, Department of Internal Medicine, Kurume University, School of Medicine, Fukuoka, Japan; Department of Respiratory Medicine, Toho University Omori Medical Center, Tokyo, Japan; Department of Thoracic Oncology, Kanagawa Cancer Center, Kanagawa, Japan; Department of Thoracic Oncology, Aichi Cancer Center Hospital, Aichi, Japan; Department of Medical Oncology, Kindai University Faculty of Medicine, Osaka, Japan; Department of Respiratory Medicine, Graduate School of Medical Sciences, Kyushu University, Fukuoka, Japan; Division of Respiratory Medicine, National Hospital Organization Kyoto Medical Center, Kyoto, Japan; Department of Respiratory Medicine and Infectious Diseases, Niigata University Graduate School of Medical and Dental Sciences, Niigata, Japan; Department of Thoracic Medical Oncology, the Cancer Institute Hospital of Japanese Foundation for Cancer Research, Tokyo, Japan; Department of Respiratory Medicine, Miyagi Cancer Center, Miyagi, Japan; Respiratory Center, Matsusaka Municipal Hospital, Mie, Japan; Department of Pulmonary Medicine, Sendai Kousei Hospital, Miyagi, Japan; Department of Thoracic Oncology, Saitama Cancer Center, Saitama, Japan; Department of Respiratory Medicine, Yokohama Municipal Citizen's Hospital, Kanagawa, Japan; Division of Pulmonary Medicine, Department of Internal Medicine, Iwate Medical University School of Medicine, Iwate, Japan; Oncology Medical, Bristol Myers Squibb, Tokyo, Japan; Oncology Medical Affairs, Ono Pharmaceutical Co, Ltd, Osaka, Japan; Department of Thoracic Oncology, National Cancer Center Hospital, Tokyo, Japan; Department of Thoracic Oncology, National Cancer Center Hospital, Tokyo, Japan

**Keywords:** ipilimumab, Japan, nivolumab, non-small-cell lung carcinoma, observational study

## Abstract

**Objective:**

As first-line treatment for stage IV or recurrent non-small cell lung cancer, combination immunotherapy with nivolumab and ipilimumab, with or without chemotherapy, had demonstrated survival benefits over chemotherapy; however, data on Japanese patients are limited.

**Methods:**

LIGHT-NING was a multicenter, observational study and retrospectively collected data. In this interim analysis, we analyzed patients who received combination immunotherapy between 27 November 2020 and 31 August 2021 for the treatment status, safety objectives (treatment-related adverse events and immune-related adverse events incidences), and effectiveness objectives (objective response rate and progression-free survival) to determine the characteristics and early safety information.

**Results:**

We analyzed 353 patients, with a median follow-up of 7.1 (interquartile range, 5.0–9.7) months. Overall, 60.1 and 39.9% received nivolumab plus ipilimumab with and without chemotherapy, respectively. In these cohorts, the median age was 67 and 72 years; 10.8 and 35.5% were aged ≥75 years; 80.2 and 79.4% were male; 5.2 and 13.5% had a performance score ≥ 2; 32.1 and 27.0% developed grade 3–4 immune-related adverse events; treatment-related deaths were observed in 6 (2.8%) and 5 (3.5%) patients, respectively. Grade 3–4 immune-related adverse event incidence was the highest within the first month of treatment in both cohorts, although the immune-related adverse event risk persisted throughout. No new safety signals were observed at this interim analysis. The median progression-free survival was 6.0 (95% confidence interval, 5.2–7.6) and 5.8 (4.3–7.0) months in nivolumab plus ipilimumab with and without chemotherapy cohorts, respectively.

**Conclusions:**

LIGHT-NING offers valuable insights into combination immunotherapy for untreated patients with stage IV or recurrent non-small cell lung cancer in Japanese real-world settings.

## Teaser

The LIGHT-NING study provides real-world data on the treatment status, safety, and effectiveness of nivolumab plus ipilimumab with or without chemotherapy, as a first-line treatment for Japanese patients with non-small cell lung cancer.

## Introduction

Lung cancer is one of the most common malignancies worldwide and is the leading cause of cancer-related deaths globally. According to the latest estimates, lung cancer accounted for 18% of all cancer-related deaths worldwide in 2020, with 2.2 million new cases and 1.8 million deaths (1). In Japan, a similar trend was observed; lung cancer accounted for ~20% of all cancer-related deaths (2). According to epidemiological statistics, > 80% of lung cancer cases are classified as non-small cell lung cancer (NSCLC) (3,4).

Historically, platinum-based doublet chemotherapy has been the standard first-line treatment for patients with advanced-stage NSCLC, particularly those without targetable epidermal growth factor receptor (EGFR) or anaplastic lymphoma kinase (ALK) genetic alterations (5–7). However, more recently, immunotherapy targeting the programmed death-1 (PD-1) checkpoint pathway has provided a therapeutic option for patients, with improved clinical outcomes compared with standard chemotherapy regimens (8).

Nivolumab, a fully human PD-1 antibody, and ipilimumab, a fully human cytotoxic T-lymphocyte associated protein 4 (CTLA-4) antibody, are immune checkpoint inhibitors (ICIs) with distinct but complementary mechanisms of action. Nivolumab restores antitumor T-cell function (9–11), while ipilimumab induces *de novo* antitumor T-cell responses, including an increase in the number of memory T cells (12–14). A combination of nivolumab and ipilimumab has shown durable survival benefits in patients with metastatic NSCLC and several other advanced solid tumors, including melanoma, renal cell carcinoma, unresectable malignant pleural mesothelioma and esophageal squamous cell carcinoma (15–19). In patients with NSCLC, the benefit of combination immunotherapy as a first-line therapy has been demonstrated using a regimen of nivolumab plus ipilimumab in CheckMate 227 (20), and in addition to two cycles of chemotherapy in CheckMate 9LA (21). Based on the results of these studies (20,21), the regimens were approved in Japan on 27 November 2020.

Owing to the strict inclusion criteria of phase III trials, the external validity of their results is limited. Therefore, it is important to conduct observational studies to evaluate safety and effectiveness in a large population in real-world settings (22–24). Similar to other phase III trials, CheckMate 9LA and CheckMate 227 excluded patients with poor Eastern Cooperative Oncology Group performance status (ECOG PS) scores, along with other patients with characteristics commonly encountered in real-world clinical settings (20,21). Moreover, long-term follow-up (25,26) and subpopulation analyses in Asian patients (27,28) from CheckMate 9LA and CheckMate 227 have been reported; however, data on the Japanese population are limited (29,30). Analyses in Asian populations are crucial to determine optimal treatment options owing to previously reported differences, such as Asians having better treatment outcomes for NSCLC than non-Asians, possibly attributable to inherent genetic variations (31). Regarding safety information, Asians should be more careful about immune-related adverse events (irAEs), especially interstitial lung disease (ILD), because Asians reportedly have a higher incidence of irAEs than Caucasians (32–34). Therefore, data on the administration of nivolumab plus ipilimumab combined with or without chemotherapy in real-world settings need to be examined.

In the present observational study, LIGHT-NING, we aimed to describe the treatment status, safety, and effectiveness of nivolumab plus ipilimumab with or without chemotherapy as a first-line therapy for untreated patients with stage IV or recurrent NSCLC under clinical conditions in Japan. In the first analysis, we reported on the background and very early safety of patients treated with nivolumab plus ipilimumab, using data from 57 patients with a follow-up duration of 5.8 (interquartile range, 4.1–6.9) months (35). Herein, we report the results of the second interim analysis of the LIGHT-NING study and offer insights into the characteristics and early safety information for this cohort in real-world clinical settings.

## Patients and methods

### Study design and treatment

In this multicenter, non-interventional, observational study, we collected data, including demographic information, information from the diagnosis of NSCLC to the initiation of treatment, treatment status, tumor response assessments and adverse events (AEs), using medical records from 48 institutions in Japan. We enrolled patients who received nivolumab plus ipilimumab with or without chemotherapy as a first-line treatment from 27 November 2020 (the date when nivolumab plus ipilimumab with or without chemotherapy was approved) to 31 December 2021. The treatment regimen was independently determined at the physicians’ discretion.

This study was registered at UMIN-CTR under identifier number UMIN000044375 and at ClinicalTrials.gov under identifier number NCT05161325. This report was based on the results of an interim analysis using data fixed by 10 June 2022, for patients who initiated treatment by 31 August 2021, with a minimum follow-up of 90 days. Data were also collected from patients who died or were lost to follow-up in <90 days.

### Ethics

This study was approved by the ethics committee at each hospital and was conducted in compliance with the Japanese Ethical Guidelines for Medical and Health Research Involving Human Subjects (36) and the Act on the Protection of Personal Information. All study procedures were conducted in accordance with the principles of the World Medical Association Declaration of Helsinki. Written informed consent was obtained from each patient before data collection; however, in cases where written informed consent from a patient or legally acceptable representatives could not be obtained, the patient was registered in an opt-out format.

### Patients

Patients aged ≥ 20 years, with histologically confirmed non-squamous or squamous, stage IV or recurrent NSCLC, with no previous systemic anticancer therapy, were eligible. We excluded patients with EGFR mutations or known ALK translocations sensitive to targeted therapy, and those who received nivolumab plus ipilimumab and added chemotherapy from the second cycle onward.

### Objectives

Details of the study objectives are presented in Supplementary Table 1. In this second interim analysis, we examined the administration status, including treatment duration, safety objectives (incidence of National Cancer Institute Common Terminology Criteria for Adverse Events Grading System [NCI-CTCAE] v 5.0 (37) grade ≥ 3 treatment-related AEs [TRAEs] and irAEs, incidence of TRAEs and irAEs leading to treatment discontinuation, treatment-related deaths, time to onset of irAEs, and time to symptom improvement) and effectiveness objectives (objective response rate [ORR], disease control rate [DCR], and progression-free survival [PFS]).

The severity of AEs was assessed according to NCI-CTCAE v 5.0 (37). Tumor evaluation was performed by each physician using images taken under clinical practice according to the methodology described in the Response Evaluation Criteria in Solid Tumors v 1.1 (38). The time to onset of AEs was defined as the time from the initiation of treatment to the onset of an AE for which the worst grade was 3 or 4. PFS was defined as the time from the date of the first nivolumab administration to the date of disease progression or death. The ORR was defined as the proportion of patients achieving complete response (CR) or partial response (PR), while the DCR was defined as the proportion of patients achieving CR, PR, or stable disease. The duration of response was defined as the time from the date of the first documented PR or CR after the initiation of nivolumab plus ipilimumab with or without chemotherapy to the date of the first documented progressive disease or death.

### Statistical analyses

Because this is a descriptive study of patients who initiated nivolumab plus ipilimumab with or without chemotherapy in the early period after approval, formal sample size calculations were not applicable, and the target sample size was set at 500 patients based on the feasibility.

The safety objectives and administration status were examined in the safety population, excluding patients with an ethical violation or patients with a short follow-up for whom no safety information was reported. The effectiveness objectives were examined in the effectiveness population, excluding patients with deviations from the protocol. Details of the safety and effectiveness population are presented in Supplementary Table 2. Data are presented as the median and range/interquartile range for continuous variables and the number (percentage) for categorical variables. The Kaplan–Meier method was performed to estimate median PFS and OS, with 95% confidence intervals (CIs) calculated using the Brookmeyer–Crowley method. Subgroup analyses for safety and effectiveness outcomes were performed according to patient backgrounds (tumor programmed death ligand 1 [PD-L1] expression, age, ECOG PS score, and histology). To examine the associations between patient background, laboratory values, and the development of grade 3–4 irAEs, odds ratios were calculated using univariate and multivariate logistic regression analyses, and *P* values were calculated using the Wald test. Variables included in the multivariable analyses were selected based on the results of the univariate analyses (*P* < 0.10) and clinical relevance (lymphocyte count). A two-sided *P* value of < 0.05 denoted statistical significance. Statistical analysis was performed using SAS v. 9.4 (SAS Institute Inc, Cary, NC, USA).

## Results

### Patients and treatment

Of the 359 patients enrolled in the present study, 353 were included in the safety population and 342 were included in the effectiveness population (Supplementary Fig. 1). The median follow-up duration was 7.1 (interquartile range, 5.0–9.7) months. The baseline characteristics of the patients are presented in Table 1. Overall, the data of 353 patients were analyzed, including 212 (60.1%) who received nivolumab plus ipilimumab with chemotherapy and 141 (39.9%) who received nivolumab plus ipilimumab. In the cohorts of patients receiving nivolumab plus ipilimumab with chemotherapy and nivolumab plus ipilimumab, the median age was 67 (range, 37–83) and 72 (range, 31–87) years, 23 (10.8%) and 50 (35.5%) patients were aged ≥ 75 years, 170 (80.2%) and 112 (79.4%) were male, and 11 (5.2%) and 19 (13.5%) had an ECOG PS score of ≥ 2, respectively. Regarding histological type, in the cohorts of patients receiving nivolumab plus ipilimumab with and without chemotherapy, 146 (68.9%) and 76 (53.9%) patients presented with adenocarcinoma, and 47 (22.2%) and 46 (32.6%) presented with squamous cell carcinoma, respectively. The clinical stages observed in the cohorts of patients receiving nivolumab plus ipilimumab with and without chemotherapy were stage IV in 176 (83.0%) and 100 (70.9%) patients, recurrence after surgery in 30 (14.2%) and 35 (24.8%), and recurrence after chemoradiation therapy in 6 (2.8%) and 6 (4.3%), respectively. Most of the patients had metastasis (*n* = 186, 87.7%; *n* = 110, 78.0%). Tumor PD-L1 expression levels were < 1% in 99 (46.7%) and 64 (45.4%) patients; 1–49% in 67 (31.6%) and 56 (39.7%); and ≥ 50% in 19 (9.0%) and 10 (7.1%), respectively. Among the patients in the two cohorts, there were 1 (0.5%) and 3 (2.1%) patient(s) with a history of ILD, 2 (0.9%) and 1 (0.7%) with concomitant ILD, and 8 (3.8%) and none with concomitant autoimmune diseases, respectively.

**Table 1:** Baseline characteristics by regimens

	Overall	Nivolumab plus ipilimumab with chemotherapy	Nivolumab plus ipilimumab
	*N* = 353	*N* = 212/353 (60.1%)	*N* = 141/353 (39.9%)
Age^a^, median (range), years	70 (31–87)	67 (37–83)	72 (31–87)
< 75	263 (74.5)	180 (84.9)	83 (58.9)
≥ 75	73 (20.7)	23 (10.8)	50 (35.5)
Sex, *n* (%)			
Male	282 (79.9)	170 (80.2)	112 (79.4)
Female	71 (20.1)	42 (19.8)	29 (20.6)
ECOG PS^b^, *n* (%)			
0–1	313 (88.7)	200 (94.3)	113 (80.1)
≥ 2	30 (8.5)	11 (5.2)	19 (13.5)
Smoking status, *n* (%)			
Never	30 (8.5)	18 (8.5)	12 (8.5)
Former	215 (60.9)	127 (59.9)	88 (62.4)
Current	108 (30.6)	67 (31.6)	41 (29.1)
Histology, *n* (%)			
Adenocarcinoma	222 (62.9)	146 (68.9)	76 (53.9)
Squamous	93 (26.3)	47 (22.2)	46 (32.6)
Other	38 (10.8)	19 (9.0)	19 (13.5)
Clinical stage, *n* (%)			
IV	276 (78.2)	176 (83.0)	100 (70.9)
Recurrence after surgery	65 (18.4)	30 (14.2)	35 (24.8)
Recurrence after chemoradiation therapy	12 (3.4)	6 (2.8)	6 (4.3)
Radiation, *n* (%)			
Definitive	49 (13.9)	22 (10.4)	27 (19.1)
Without durvalumab	19 (5.4)	11 (5.2)	8 (5.7)
With durvalumab	20 (5.7)	9 (4.2)	11 (7.8)
Palliative	58 (16.4)	34 (16.0)	24 (17.0)
Metastasis^c^, *n* (%)	296 (83.9)	186 (87.7)	110 (78.0)
Bone	104 (29.5)	63 (29.7)	41 (29.1)
Liver	40 (11.3)	26 (12.3)	14 (9.9)
Brain	64 (18.1)	42 (19.8)	22 (15.6)
Other^d^	224 (63.5)	143 (67.5)	81 (57.4)
Tumor PD-L1 expression^e^, *n* (%)			
< 1%	163 (46.2)	99 (46.7)	64 (45.4)
1–49%	123 (34.8)	67 (31.6)	56 (39.7)
≥ 50%	29 (8.2)	19 (9.0)	10 (7.1)
Unknown	30 (8.5)	22 (10.4)	8 (5.7)
Interstitial lung disease, *n* (%)			
None	346 (98.0)	209 (98.6)	137 (97.2)
Past	4 (1.1)	1 (0.5)	3 (2.1)
Current	3 (0.8)	2 (0.9)	1 (0.7)
Autoimmune disease, *n* (%)	8 (2.3)	8 (3.8)	0 (0)

ECOG PS, Eastern Cooperative Oncology Group performance status; PD-L1, programmed cell death ligand 1.

^a^Data were unknown for 17 patients.

^b^Data were unknown for 10 patients.

^c^Multiple choices possible.

^d^Other includes pleural dissemination (*n* = 84/353, 23.8%), distant lymph nodes (*n* = 63/353, 17.8%), contralateral lung (*n* = 59/353, 16.7%) and so on.

^e^Data were missing for 8 patients.

Table 2 presents the treatment patterns by regimens. By 3 months, 7 (3.3%) and 12 (8.5%) patients discontinued ipilimumab alone, and 100 (47.2%) and 74 (52.5%) patients discontinued both nivolumab and ipilimumab in the cohorts of patients who received nivolumab plus ipilimumab with and without chemotherapy, respectively. Regarding chemotherapy, 162 (76.4%) patients received two cycles of treatment in the nivolumab plus ipilimumab with chemotherapy cohort.

**Table 2:** Treatment patterns by regimens

	Overall	Nivolumab plus ipilimumab with chemotherapy	Nivolumab plus ipilimumab
	*N* = 353	*N* = 212	*N* = 141
Treatment discontinuation by 3 months, *n* (%)			
Ipilimumab	19 (5.4)	7 (3.3)	12 (8.5)
Nivolumab and ipilimumab	174 (49.3)	100 (47.2)	74 (52.5)
Cycles of chemotherapy received, *n* (%)			
One cycle	50 (14.2)	50 (23.6)	-
Two cycles	162 (45.9)	162 (76.4)	-
Patients received local treatment, (%)			
Surgery	2 (0.6)	0 (0)	2 (1.4)
Radiotherapy	34 (9.6)	17 (8.0)	17 (12.1)

### Safety

Table 3 summarizes the TRAEs and irAEs in both cohorts. In the nivolumab plus ipilimumab with chemotherapy cohort, grade 3–4 TRAEs were observed in 94 (44.3%) patients and grade 3–4 irAEs in 68 (32.1%); any grade TRAEs and irAEs leading to discontinuation of nivolumab and/or ipilimumab were observed in 81 (38.2%) and 61 (28.8%) patients, respectively. In the nivolumab plus ipilimumab cohort, grade 3–4 TRAEs and irAEs were observed in 38 (27.0%) patients; any grade irAEs leading to discontinuation of nivolumab, ipilimumab and/or chemotherapy were observed in 44 (31.2%) patients. In the nivolumab plus ipilimumab with chemotherapy cohort, treatment-related deaths were observed in 6 (2.8%) patients (pneumonitis in 3 [1.4%], hepatitis in 1 [0.5%], bronchopulmonary aspergillosis in 1 [0.5%], and septic shock in 1 [0.5%]). In the nivolumab plus ipilimumab cohort, treatment-related deaths were observed in 5 (3.5%) patients (pneumonitis in 4 [2.8%] and sepsis in 1 [0.7%]).

**Table 3:** Summary of safety

	Overall			Nivolumab plus ipilimumab with chemotherapy			Nivolumab plus ipilimumab	
	*N* = 353			*N* = 212			*N* = 141	
	Any grade	Grade 3–4		Any grade	Grade 3–4		Any grade	Grade 3–4
Grade 3–4 TRAEs, *n* (%)	-	132 (37.4)		-	94 (44.3)		-	38 (27.0)
Grade 3–4 irAEs, *n* (%)	-	106 (30.0)		-	68 (32.1)		-	38 (27.0)
TRAEs leading to discontinuation, *n* (%)								
All components of the regimen	63 (17.8)	36 (10.2)		28 (13.2)	15 (7.1)		35 (24.8)	21 (14.9)
Any component of the regimen	125 (35.4)	74 (21.0)		81 (38.2)	48 (22.6)		44 (31.2)	26 (18.4)
irAE leading to discontinuation, *n* (%)								
All components of the regimen	61 (17.3)	33 (9.3)		26 (12.3)	12 (5.7)		35 (24.8)	21 (14.9)
Any component of the regimen	105 (29.7)	61 (17.3)		61 (28.8)	35 (16.5)		44 (31.2)	26 (18.4)
Treatment-related deaths, *n* (%)	11 (3.1)			6 (2.8)			5 (3.5)	

irAE, immune-related adverse event; TRAE, treatment-related adverse event.

Details of the grade 3–4 irAEs and irAEs that led to discontinuation of any treatment component are provided in Supplementary Tables 3 and 4. The most frequent grade 3–4 irAEs were rashes or dermatitis in 18 (8.5%) patients in the nivolumab plus ipilimumab with chemotherapy cohort and hepatitis in 11 (7.8%) and pneumonitis in 11 (7.8%) patients in the nivolumab plus ipilimumab cohort. The most frequent grade 3–4 irAEs leading to treatment discontinuation were pneumonitis in 9 (4.2%) patients in the nivolumab plus ipilimumab with chemotherapy cohort and hepatitis in 10 (7.1%) patients in the nivolumab plus ipilimumab cohort. Summaries of irAEs and TRAEs for both cohorts according to age and ECOG PS scores are presented in Supplementary Tables 5 and 6.

**Figure 1:**
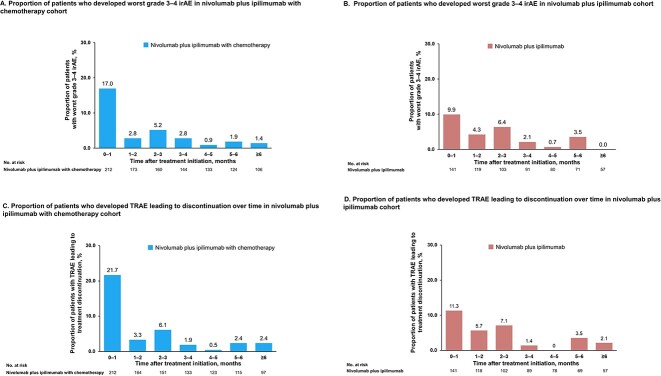
Proportion of patients who developed CTCAE grade 3–4 irAEs or TRAEs leading to discontinuation over time. Proportions of patients who developed at least one irAE for which the worst grade was 3–4 within each time interval in the nivolumab plus ipilimumab with (A) and without (B) chemotherapy cohorts. The proportion of patients who developed at least one TRAE leading to treatment discontinuation within each time interval in the nivolumab plus ipilimumab with (C) and without (D) chemotherapy cohorts. CTCAE, Common Terminology Criteria for Adverse Events Grading System; irAE, immune-related adverse event; TRAE, treatment-related adverse event.

The proportion of patients who developed at least one irAE for which the worst grade was 3 or 4 within each time interval according to the cohorts is shown in Fig. 1A and B. In both cohorts, the incidence of grade 3–4 irAEs was highest within the first month of treatment. The proportion of patients who developed at least one TRAE leading to the discontinuation of any treatment component within each time interval according to the cohorts is shown in Fig. 1C and D. Most TRAEs leading to discontinuation occurred within the first month after treatment initiation.

**Figure 2:**
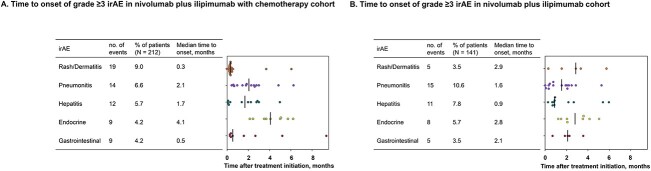
Time to onset of CTCAE grade ≥ 3 irAEs. Time from treatment initiation to onset of CTCAE grade ≥ 3 irAEs in the nivolumab plus ipilimumab with (A) and without (B) chemotherapy cohorts. The figures show grade ≥ 3 irAEs with an incidence of ≥1% in both cohorts. Vertical lines indicate the median to onset. Time to onset of an irAE was defined as the time from the initiation of treatment to the onset of an irAE for which the worst grade was ≥ 3. CTCAE, Common Terminology Criteria for Adverse Events Grading System; irAE, immune-related adverse event.

The time to onset of grade ≥ 3 irAEs according to the cohorts is shown in Fig. 2A and B. Grade ≥ 3 irAEs occurred throughout the follow-up period, particularly within the first month after the treatment initiation. These data suggest that the incidence of irAEs was the highest in the early stages of administration, although the risk of various irAEs existed throughout the observation period.

Supplementary Table 7 presents the results of the univariate and multivariate analyses of associations among patient backgrounds, laboratory values and grade ≥ 3 irAEs overall. No independent predictors of grade ≥ 3 irAEs were found.

### Effectiveness

**Figure 3:**
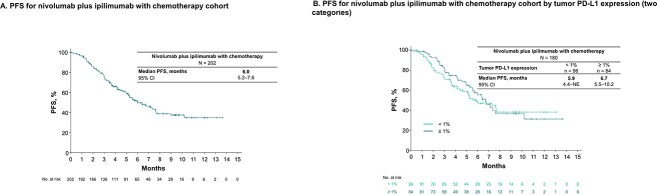
Kaplan–Meier analysis of PFS in the nivolumab plus ipilimumab with chemotherapy cohort. Kaplan–Meier analysis of PFS in the nivolumab plus ipilimumab with chemotherapy cohort (A) and that according to tumor PD-L1 expression levels of two categories (<1 and ≥1%) (B). CI, confidence interval; NE, not estimated; PD-L1, programmed death ligand 1; PFS, progression-free survival.

**Figure 4:**
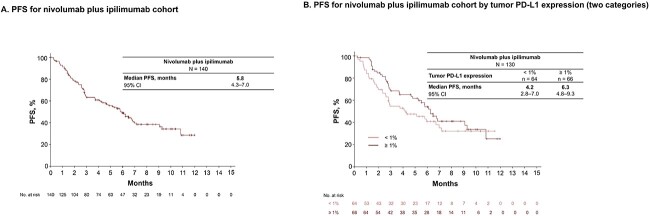
Kaplan–Meier analysis of PFS in the nivolumab plus ipilimumab cohort. Kaplan–Meier analysis of PFS in the nivolumab plus ipilimumab cohort (A) and that according to tumor PD-L1 expression levels of two categories (<1 and ≥1%) (B). CI, confidence interval; PD-L1, programmed death ligand 1; PFS, progression-free survival.

In this interim analysis, in the nivolumab plus ipilimumab with chemotherapy cohort, disease progression or death occurred in 101 (50.0%) patients, with a median PFS of 6.0 (95% CI, 5.2–7.6; Fig. 3A) months. Based on tumor PD-L1 expression, the median (95% CI) PFS was 5.9 (4.4–not estimated) months in the < 1% subgroup and 6.7 (5.5–10.2) months in the ≥ 1% subgroup (Fig. 3B). In the nivolumab plus ipilimumab cohort, disease progression or death occurred in 77 (55.0%) patients, with a median PFS of 5.8 (95% CI, 4.3–7.0; Fig. 4A) months. Based on tumor PD-L1 expression, the median (95% CI) PFS was 4.2 (2.8–7.0) months in the < 1% subgroup and 6.3 (4.8–9.3) months in the ≥ 1% subgroup (Fig. 4B).

Table 4 presents the ORR assessed by each physician. In the nivolumab plus ipilimumab with chemotherapy cohort (*n* = 149), the ORR was 39.6% (95% CI, 31.7–47.9), with 1 (0.7%) patient achieving CR and 58 (38.9%) achieving PR. In the nivolumab plus ipilimumab cohort (*n* = 92), the ORR was 37.0% (95% CI, 27.1–47.7), with 2 (2.2%) patients achieving CR and 32 (34.8%) achieving PR.

**Table 4:** Objective response rates by regimens

	Overall	Nivolumab plus ipilimumab with chemotherapy	Nivolumab plus ipilimumab
	*N* = 241	*N* = 149	*N* = 92
Objective responses, *n* (%)	93 (38.6)	59 (39.6)	34 (37.0)
(95% CI)	(32.4–45.1)	(31.7–47.9)	(27.1–47.7)
Best overall response, *n* (%)			
Complete response	3 (1.2)	1 (0.7)	2 (2.2)
Partial response	90 (37.3)	58 (38.9)	32 (34.8)
Stable disease	55 (22.8)	31 (20.8)	24 (26.1)
Progressive disease	84 (34.9)	52 (34.9)	32 (34.8)
Not evaluable	9 (3.7)	7 (4.7)	2 (2.2)
Disease controls, *n* (%)	148 (61.4)	90 (60.4)	58 (63.0)
(95% CI)	(54.9–67.6)	(52.1–68.3)	(52.3–72.9)

The number of patients was the number for whom the results of the best overall response were available. CI, confidence interval.

Subgroup analyses showed that in the nivolumab plus ipilimumab with chemotherapy cohort, the median (95% CI) PFS was 5.7 (4.9–7.4) months in the <75 years subgroup, not reached (5.1–not estimated) in the ≥75 years subgroup, 6.7 (5.3–7.7) months in the ECOG PS 0–1 subgroup, 2.2 (1.6–5.3) months in the ECOG PS ≥2 subgroup, 5.3 (4.9–7.7) months in the squamous cell carcinoma subgroup and 6.4 (4.7–7.6) months in the adenocarcinoma subgroup (Supplementary Table 8). In the nivolumab plus ipilimumab cohort, the median (95% CI) PFS was 6.1 (4.1–7.2) months in the <75 years subgroup, 5.9 (3.8–not estimated) months in the ≥75 years subgroup, 6.3 (5.3–9.1) months in the ECOG PS 0–1 subgroup, 1.4 (0.8–4.8) months in the ECOG PS ≥2 subgroup, 5.9 (4.6–not estimated) months in the squamous cell carcinoma subgroup and 6.1 (2.8–9.3) months in the adenocarcinoma subgroup (Supplementary Table 9). The ORR for the subgroups is shown in Supplementary Tables 8 and 9.

## Discussion

The present study provides data on the treatment status, safety and effectiveness of dual immunotherapy combination of nivolumab plus ipilimumab with or without chemotherapy as a first-line therapy for untreated patients with stage IV or recurrent NSCLC under clinical conditions in Japan. Dual immunotherapy is expected to have therapeutic effects; nonetheless, appropriate management is necessary because TRAEs and irAEs often occur in the early stages of administration. This interim analysis was conducted to provide early safety data with practical utility, despite a short median follow-up period (7.1 months).

The present study enrolled a wide variety of patients from real-world settings, including those who were infrequently enrolled (aged ≥75 years) or not eligible for pivotal trials due to, for example, a poor ECOG PS score, a history of ILD, a history of autoimmune disease, or previously treated with durvalumab, a treatment for stage III NSCLC). The proportion of patients aged ≥75 years, having an ECOG PS score of ≥2, squamous cell carcinoma, and PD-L1 expression <1% was higher in the present study than in the CheckMate 9LA (20) and CheckMate 227 (21) phase III trials. In the present study, differences in patient backgrounds according to the regimens were observed: age ≥ 75 years in 10.8 and 35.5%, an ECOG PS score of ≥2 in 5.2 and 13.5%, and squamous cell carcinoma in 22.2 and 32.6% of patients in the nivolumab plus ipilimumab with and without chemotherapy cohorts, respectively. The results of this interim analysis suggest that physicians consider patient background characteristics to select whether chemotherapy is used with the dual immunotherapy regimen. Therefore, the safety and efficacy of nivolumab plus ipilimumab with or without chemotherapy could not be directly compared in this study and should be further studied after adjusting for differences in patient background.

No new safety signals were observed, consistent with the results of CheckMate 9LA (20) and CheckMate 227 (21). The incidence of grade ≥ 3 TRAEs in the present study was in line with the incidence rates in CheckMate 9LA (20) and CheckMate 227 (21). The frequency of irAEs was greatest in the first month after treatment initiation, although the risk of various irAEs existed even after >6 months. During treatment with dual immunotherapy regimens, patients should be closely monitored for the development and management of irAEs, especially during the first few treatment cycles, as reported in a previous pooled analysis of clinical trials (39) and the present study.

A higher than anticipated rate of treatment-related deaths with nivolumab plus ipilimumab treatment was observed in the NIPPON study (40,41). The NIPPON study was a randomized, controlled phase III trial designed to compare nivolumab plus ipilimumab with 2 cycles of chemotherapy (same regimen as CheckMate 9LA (20)) versus pembrolizumab plus 4 cycles of chemotherapy for treatment-naive patients with metastatic NSCLC. Of the 48 participating institutions in LIGHT-NING, ~60% were also participating in the NIPPON study. Patient enrollment in the NIPPON study started in April 2021. Deaths reported as treatment-related occurred in 7.4% (11/148) patients randomized to nivolumab plus ipilimumab with 2 cycles of chemotherapy. Deaths were attributed to pneumonitis in 4, cytokine release syndrome in 3, sepsis in 1, hemophagocytic syndrome in 1, and myocarditis in 2 patient(s). Although it is difficult to compare the differences in the incidences of treatment-related death between the NIPPON study and other studies, the following data are noteworthy: Seven (2%) deaths occurred in the nivolumab plus ipilimumab with chemotherapy arm of CheckMate 9LA (20) owing to acute kidney failure attributed to chemotherapy, diarrhea, hepatotoxicity, hepatitis, pneumonitis, sepsis with acute renal insufficiency, and thrombocytopenia (in one patient each); eight (1.4%) deaths were attributed to nivolumab plus ipilimumab by pneumonitis (in 4 patients), and shock, myocarditis, acute tubular necrosis, and cardiac tamponade (in 1 patient each) in the the nivolumab plus ipilimumab arm of CheckMate 227 (21). These rates were similar to those in the current study, where six (2.8%) deaths (pneumonitis in 3 [1.4%], hepatitis in 1 [0.5%], bronchopulmonary aspergillosis in 1 [0.5%], and septic shock in 1 [0.5%]) occurred in the nivolumab plus ipilimumab with chemotherapy cohort; and five (3.5%) deaths (pneumonitis in 4 [2.8%], and sepsis in 1 [0.7%]) occurred in the nivolumab plus ipilimumab cohort. These safety results are consistent with the FINN study, which is a real-world study with 90 sites and 650 patients planned to evaluate the combination of nivolumab plus ipilimumab with 2 cycles of chemotherapy in patients with metastatic NSCLC in clinical practice in Germany. At the interim analysis of the ongoing FINN study, 256 patients were included in the interim analysis with a minimum follow-up of 5.6 months (42). TRAEs were reported in 53.9% of patients. Severe TRAEs were reported in 17.6% of patients. There was 1 treatment-related death from immune-mediated hepatitis. In CheckMate 9LA, CheckMate 227, NIPPON, and LIGHT-NING, pneumonitis was the leading cause of treatment-related deaths, demonstrating a similar safety profile between clinical trials and real-world settings. The frequency of PD-1/PD-L1 inhibitor-induced ILD varies among reports, and the frequency of any grade ILDs is generally <10% (24,43–45). However, the frequency of ILD is speculated to be higher in Japan (32); this is an important point to consider because it can be fatal in ~10% of patients (46,47). Therefore, careful patient monitoring in pneumonitis and early irAE managament when it develops in pneumonitis are necessary.

There is a paucity of studies assessing the risk factors for irAEs for dual immunotherapy with nivolumab and ipilimumab. A previous systematic review and meta-analysis using the results of 25 trials evaluated risk factors related to the development of irAEs, including baseline characteristics and laboratory markers (48). In this systematic review and meta-analysis, the most frequently reported risk factors for the development of irAEs were sex (19 studies), ECOG PS (16 studies), histology (16 studies), smoking (13 studies), and age (12 studies) (48). In this study, no risk factors for the development of grade ≥ 3 irAEs were found. Longer follow-up with enrollment of additional patients may help in identifying any risk factors for grade ≥ 3 irAEs.

Despite a limited sample size, the CheckMate 9LA (28) and CheckMate 227 (27) trials reported that the efficacy of nivolumab plus ipilimumab with and without chemotherapy in Asians was similar to that of the global population. However, only a subset of Asian patients were Japanese, and data on Japanese patients in this setting are generally limited (29,30). This study described the effectiveness of the dual immunotherapy treatment regimen with a relatively large sample size in real-world settings. For nivolumab plus ipilimumab with chemotherapy, the median PFS was 6.0 months in this study (ORR = 39.6%) and 6.7 months in CheckMate 9LA (ORR = 38.2%) (20). For nivolumab plus ipilimumab without chemotherapy, the median PFS was 5.8 months in this study (ORR = 37.0%) and 5.1 months in CheckMate 227 (ORR = 33.1%) (21). The original concept of CheckMate 9LA (20) in the first place was to help address early disease progression by adding only 2 cycles of chemotherapy early in CheckMate 227 regimen (21). Therefore, if the response rates are similar between the two regimens, it indicates that patients requiring first-line chemotherapy may be rescued by only 2 cycles of chemotherapy, and the long-term efficacy is expected to be similar thereafter. With the enrollment of additional patients and long-term follow-up data, an analysis that considers differences in patient backgrounds by regimen and the significance of the addition of chemotherapy in terms of efficacy will be conducted in LIGHT-NING.

This study has certain limitations. First, owing to the observational nature of the study, the use of chemotherapy in addition to nivolumab plus ipilimumab relied on the physician’s judgment, which potentially introduced selection bias. Second, data for this study were obtained from medical records; therefore, any information that was not recorded was treated as missing data, and evaluations of the effectiveness and safety objectives were based on examinations for which the methodology and frequency were independently determined by the physicians. Third, regarding safety objectives, this study collected data on grade ≥ 3 irAEs and any grade of irAEs leading to treatment discontinuation; thus, this study could not adequately examine grade 1–2 irAEs that did not lead to treatment discontinuation. Fourth, ORRs and PFS were not assessed under pre-specified clinical trial criteria; therefore, an independent central review was missing. As a result, endpoint differences owing to variations in the methodology and frequency among physicians in trials versus real-world practice, including in the radiological evaluation of tumor response, could limit the comparison of these real-world data to those from clinical trials. Finally, because this is the interim analysis, the follow-up period and number of enrolled patients were limited. As frequently seen in other studies, the interpretations of subgroup analyses according to age, ECOG PS score, PD-L1 expression and histology may be limited owing to the small number of patients. Future follow-up studies will reveal the safety and effectiveness in real-world clinical settings with greater precision.

In conclusion, through an interim analysis, this study provides Japanese real-world treatment status, safety and effectiveness of nivolumab plus ipilimumab with or without chemotherapy, as a first-line therapy for untreated patients with stage IV or recurrent NSCLC. No new safety signals were observed, with a similar trend of safety profile as in CheckMate 227 and CheckMate 9LA. The incidence of irAEs was highest in the early stages of administration, although the risk of various irAEs existed throughout the observation period.

## Supplementary Material

JJCO-23-0617R1_Imai_et_al_LIGHT-NING_JJCO_Supplementary_Figure_1_hyad195

Clean_edited2_jjco-23-0617r1_imai_et_al_light-ning_jjco_supplementary_tables_hyad195

## Data Availability

Bristol Myers Squibb policy on data sharing may be found at https://www.bms.com/researchers-and-partners/independent-research/data-sharing-request-process.html.

## List of Abbreviations

AE, adverse event; ALK, anaplastic lymphoma kinase; CI, confidence interval; CR, complete response; CTLA-4, cytotoxic T-lymphocyte associated protein 4; DCR, disease control rate; ECOG PS, Eastern Cooperative Oncology Group performance status; EGFR, epidermal growth factor receptor; ICI, immune checkpoint inhibitor; ILD, interstitial lung disease; irAE, immune-related adverse event; NCI-CTCAE, National Cancer Institute Common Terminology Criteria for Adverse Events Grading System; NSCLC, non-small cell lung cancer; ORR, objective response rate; PD-1, programmed death-1; PD-L1, programmed death ligand 1; PFS, progression-free survival; PR, partial response; TRAE, treatment-related adverse event.
