# Correction to: First-line nivolumab plus ipilimumab with or without chemotherapy for Japanese patients with non-small cell lung cancer: LIGHT-NING study

**DOI:** 10.1093/jjco/hyae069

**Published:** 2024-05-22

This is a correction to: Hisao Imai, Takashi Kijima, Koichi Azuma, Kazuma Kishi, Haruhiro Saito, Teppei Yamaguchi, Junko Tanizaki, Yasuto Yoneshima, Kohei Fujita, Satoshi Watanabe, Satoru Kitazono, Tatsuro Fukuhara, Osamu Hataji, Yukihiro Toi, Hideaki Mizutani, Yusuke Hamakawa, Makoto Maemondo, Tomoyuki Ohsugi, Keisuke Suzuki, Hidehito Horinouchi, Yuichiro Ohe, First-line nivolumab plus ipilimumab with or without chemotherapy for Japanese patients with non-small cell lung cancer: LIGHT-NING study, Japanese Journal of Clinical Oncology, Volume 54, Issue 4, April 2024, Pages 452–462, https://doi.org/10.1093/jjco/hyad195

In the originally published version of this manuscript, there was errored text needing correction.

In section Patients and methods, subheading Objectives, in the second sentence: “[...]immune-related AEs,[...]” should read “irAEs”.

In Table 1, beneath “Radiation” the labels “with durvalumab” and “without durvalumab” were erroneously transposed. These are now reversed. In the legend to Table 1: “[...]programmed cell death 1–ligand 1.” is corrected to read: “[...]programmed cell death ligand 1.”

The beginning of the legend to Figure 1 should read: “Proportion of patients who developed grade CTCAE 3–4 irAEs[...]” instead of: “Proportion of patients who developed grade 3–4 irAEs[...]”. The fourth sentence should read: “CTCAE, Common Terminology Criteria for Adverse Events Grading System;[...]” instead of: “CTCAE, common terminology criteria for adverse events;[...]”.

The last sentence of the legend to Figure 2 should read: “CTCAE, Common Terminology Criteria for Adverse Events Grading System; irAE, immune-related adverse event.” instead of: “irAE, immune-related adverse event.”

In section **Results**, under subheading **Safety**, the last paragraph should read: “Supplementary Table 7 presents the results of the univariate and multivariate analyses of associations among patient backgrounds, laboratory values and grade ≥ 3 irAEs overall. No independent predictors of grade ≥ 3 irAEs were found.” instead of: “Supplementary Table 7 presents the results of the univariate and multivariate analyses of associations among patient backgrounds, laboratory values and grade 3–4 irAEs overall. No independent predictors of grade 3–4 irAEs were found.”

The authors believe that these corrections do not change their interpretation of the results.

The above errors and abbreviations have been corrected in the article.

