# Supplementary figures and images for: First-line nivolumab plus ipilimumab with or without chemotherapy for Japanese patients with non-small cell lung cancer: LIGHT-NING study
Source: Jpn J Clin Oncol. 2024 Jan 25;54(4):452–62. doi: 10.1093/jjco/hyad195 (PMC10999773; doi:10.1093/jjco/hyad195)

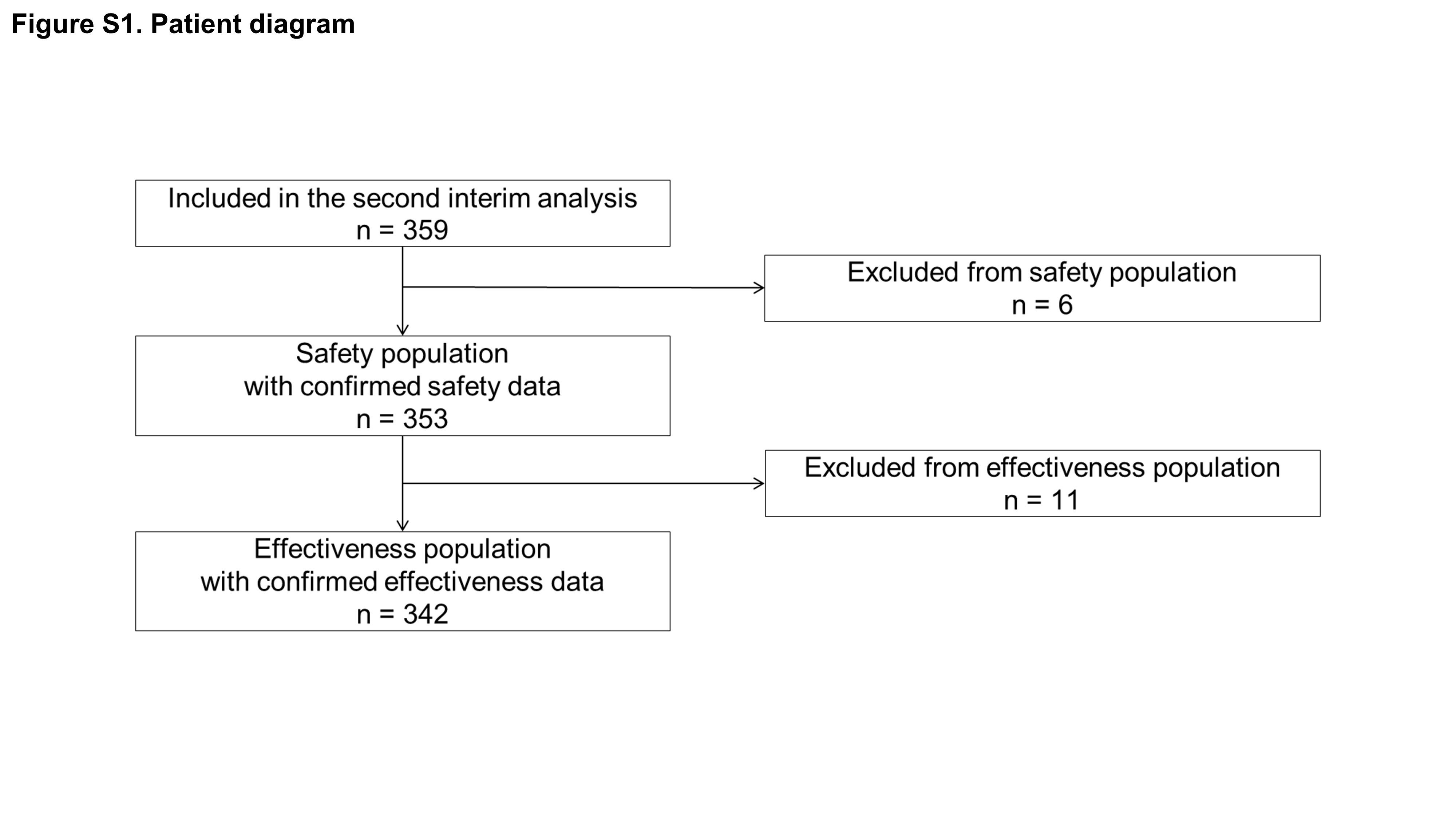

Supplement: JJCO-23-0617R1_Imai_et_al_LIGHT-NING_JJCO_Supplementary_Figure_1_hyad195 [file jjco-23-0617r1_imai_et_al_light-ning_jjco_supplementary_figure_1_hyad195.jpeg]
