# Supplementary material for: First-line nivolumab plus ipilimumab with or without chemotherapy for Japanese patients with non-small cell lung cancer: LIGHT-NING study
Source: Jpn J Clin Oncol. 2024 Jan 25;54(4):452–62. doi: 10.1093/jjco/hyad195 (PMC10999773; doi:10.1093/jjco/hyad195)
Supplement: Clean_edited2_jjco-23-0617r1_imai_et_al_light-ning_jjco_supplementary_tables_hyad195 [file clean_edited2_jjco-23-0617r1_imai_et_al_light-ning_jjco_supplementary_tables_hyad195.docx]

Supporting information

Supplementary Table 1. Study objectives

| Primary | - Descriptions of administration (duration of treatment, rates of patients with second-line treatment, etc.) - Effectiveness (OS, TNT, TFS, and treatment continuation rate) - Safety (incidence of CTCAE v 5.0 grade ≥ 3 irAEs and incidence of TRAEs leading to treatment discontinuation) |
| --- | --- |
| Secondary | - Effectiveness in patients evaluated for response in accordance with RECIST v 1.1 (PFS, ORR, DCR, and DoR) - Effectiveness and safety by patient background - Effectiveness and safety of nivolumab plus ipilimumab with or without chemotherapy by adjustment for confounding factors - Time to onset of irAEs, treatment for irAEs, time to symptom improvement, and impact on effectiveness - Descriptions of administration (duration of treatment, reasons for treatment discontinuation, etc.), effectiveness (response rate), and safety (treatment-related death) of a second-line treatment - Effectiveness in patients who discontinued treatment due to TRAEs within 90 days - Descriptions of administration (duration of treatment, reasons for treatment discontinuation, etc.), effectiveness (response rate and overall survival), and safety (treatment-related death) of second-line treatment in patients with disease progression within 90 days |

CTCAE, Common Terminology Criteria for Adverse Events; DCR, disease control rate; DoR, duration of response; irAE, immune-related adverse event; ORR, objective response rate; OS, overall survival; PFS, progression-free survival; RECIST, Response Evaluation Criteria in Solid Tumors; TFS, treatment-free survival; TRAE, treatment-related adverse event; TNT, time to next treatment.

Supplementary Table 2. Analysis population

| Safety | Of the enrolled patients, the following patients were excluded:  • Patients for whom safety data could not fixed.  • Patients who withdraw consent.  • Patients with duplicate enrollment (duplication will be excluded).  • Patients without reported adverse events and within 7 days of follow-up.  • Patients who started treatment with nivolumab plus ipilimumab and switched to nivolumab plus ipilimumab with chemotherapy after disease progression has been confirmed.^a^  • Patients who were confirmed to have participated in a clinical trial in the second-line or subsequent treatment after obtaining informed consent.^b^ |
| --- | --- |
| Effectiveness | Of the enrolled patients, the following patients were excluded:  • Patients who violated the inclusion criteria.  • Patients who violated the exclusion criteria.  • Patients who withdraw consent.  • Patients with duplicate enrollment (duplication will be excluded).  • Patients without reported adverse events and within 7 days of follow-up.  • Patients who started treatment with nivolumab plus ipilimumab and switched to nivolumab plus ipilimumab with chemotherapy after disease progression has been confirmed.^a^  • Patients who were confirmed to have participated in a clinical trial in the second-line or subsequent treatment after obtaining informed consent.^b^  • Patients who received chemotherapy with a regimen other than regimens specified in the selection criteria.^c^ |

^a^ Only information from the first-line treatment will be used for analysis.

^b^ For patients participating in a clinical trial for second-line treatment, only information at the time of first-line treatment will be used for analysis. For patients participating in a clinical trial for third-line or subsequent treatment, information up to the second-line treatment and outcome information up to the time of participation in the clinical trial will be used for analysis.

^c^ Pemetrexed plus cisplatin or carboplatin is acceptable for patients with non-squamous cell carcinoma, and paclitaxel plus carboplatin is acceptable for patients with squamous cell carcinoma.

Supplementary Table 3. Details of Grade 3–4 irAEs by regimens

|  | Overall | Nivolumab plus ipilimumab with chemotherapy | Nivolumab plus ipilimumab |
| --- | --- | --- | --- |
|  | *N* = 353 | *N* = 212 | *N* = 141 |
| Rash/Dermatitis | 23 (6.5) | 18 (8.5) | 5 (3.5) |
| Pneumonitis | 22 (6.2) | 11 (5.2) | 11 (7.8) |
| Hepatitis | 21 (5.9) | 10 (4.7) | 11 (7.8) |
| Endocrine | 17 (4.8) | 9 (4.2) | 8 (5.7) |
| Gastrointestinal disorder | 13 (3.7) | 8 (3.8) | 5 (3.5) |
| Hematological disorder | 10 (2.8) | 9 (4.2) | 1 (0.7) |
| Nephritis/Renal dysfunction | 5 (1.4) | 5 (2.4) | 0 (0) |
| Other | 12 (3.4) | 9 (4.2) | 3 (2.1) |

Presented are irAE categories observed in more than 1% of patients.

The severity of adverse events was assessed according to CTCAE v 5.0. CTCAE, National Cancer Institute Common Terminology Criteria for Adverse Events Grading System; irAE, immune-related adverse event.

Supplementary Table 4. Details of irAEs leading to discontinuation by regimens

|  | Overall | |  | Nivolumab plus ipilimumab with chemotherapy | |  | Nivolumab plus ipilimumab | |
| --- | --- | --- | --- | --- | --- | --- | --- | --- |
|  | *N* = 353 | |  | *N* = 212 | |  | *N* = 141 | |
|  | Grade 1–2 | Grade 3–4 |  | Grade 1–2 | Grade 3–4 |  | Grade 1–2 | Grade 3–4 |
| Rash/Dermatitis | 6 (1.7) | 6 (1.7) |  | 4 (1.9) | 5 (2.4) |  | 2 (1.4) | 1 (0.7) |
| Pneumonitis | 16 (4.5) | 17 (4.8) |  | 10 (4.7) | 9 (4.2) |  | 6 (4.3) | 8 (5.7) |
| Hepatitis | 4 (1.1) | 16 (4.5) |  | 2 (0.9) | 6 (2.8) |  | 2 (1.4) | 10 (7.1) |
| Endocrine | 1 (0.3) | 5 (1.4) |  | 0 (0) | 1 (0.5) |  | 1 (0.7) | 4 (2.8) |
| Gastrointestinal disorder | 6 (1.7) | 7 (2.0) |  | 5 (2.4) | 5 (2.4) |  | 1 (0.7) | 2 (1.4) |
| Hematological disorder | 0 (0) | 1 (0.3) |  | 0 (0) | 1 (0.5) |  | 0 (0) | 0 (0) |
| Nephritis/Renal dysfunction | 1 (0.3) | 2 (0.6) |  | 1 (0.5) | 2 (0.9) |  | 0 (0) | 0 (0) |
| Other | 2 (0.6) | 4 (1.1) |  | 2 (0.9) | 3 (1.4) |  | 0 (0) | 1 (0.7) |

The severity of adverse events was assessed according to CTCAE v 5.0. CTCAE, Common Terminology Criteria for Adverse Events Grading System; irAE, immune-related adverse event.

Supplementary Table 5. Summary of safety by subgroups in nivolumab plus ipilimumab with chemotherapy

|  | Age^a^ | | | |  | ECOG PS^b^ | | | |
| --- | --- | --- | --- | --- | --- | --- | --- | --- | --- |
|  | < 75 years  *N* = 180 | | ≥ 75 years  *N* = 23 | |  | 0–1  *N* = 200 | | ≥ 2  *N* = 11 | |
|  | Any grade | Grade 3–4 | Any grade | Grade 3–4 |  | Any grade | Grade 3–4 | Any grade | Grade 3–4 |
| Grade 3–4 TRAEs, n (%) | - | 80 (44.4) | - | 7 (30.4) |  | - | 90 (45.0) | - | 4 (36.4) |
| Grade 3–4 irAEs, n (%) | - | 56 (31.1) | - | 7 (30.4) |  | - | 65 (32.5) | - | 3 (27.3) |
| TRAEs leading to discontinuation, n (%) |  |  |  |  |  |  |  |  |  |
| All components of the regimen | 26 (14.4) | 14 (7.8) | 1 (4.3) | 1 (4.3) |  | 25 (12.5) | 13 (6.5) | 3 (27.3) | 2 (18.2) |
| Any components of the regimen | 67 (37.2) | 41 (22.8) | 9 (39.1) | 5 (21.7) |  | 76 (38.0) | 44 (22.0) | 5 (45.5) | 4 (36.4) |
| irAEs leading to discontinuation, n (%) |  |  |  |  |  |  |  |  |  |
| All components of the regimen | 24 (13.3) | 11 (6.1) | 1 (4.3) | 1 (4.3) |  | 23 (11.5) | 10 (5.0) | 3 (27.3) | 2 (18.2) |
| Any components of the regimen | 52 (28.9) | 31 (17.2) | 5 (21.7) | 3 (13.0) |  | 57 (28.5) | 32 (16.0) | 4 (36.4) | 3 (27.3) |
| Treatment-related deaths, n (%) | 4 (2.2) | | 1 (4.3) | |  | 6 (3.0) | | 0 (0) | |

The severity of AEs was assessed according to CTCAE v 5.0.

^a^ Data were unknown for 9 patients.

^b^ Data were unknown for 1 patient.

CTCAE, Common Terminology Criteria for Adverse Events Grading System; ECOG PS, Eastern Cooperative Oncology Group performance status; irAE, immune-related adverse event; TRAE, treatment-related adverse event.

Supplementary Table 6. Summary of safety by subgroups in nivolumab plus ipilimumab

|  | Age^a^ | | | |  | ECOG PS^b^ | | | |
| --- | --- | --- | --- | --- | --- | --- | --- | --- | --- |
|  | < 75 years  *N* = 83 | | ≥ 75 years  *N* = 50 | |  | 0–1  *N* = 113 | | ≥ 2  *N* = 19 | |
|  | Any grade | Grade 3–4 | Any grade | Grade 3–4 |  | Any grade | Grade 3–4 | Any grade | Grade 3–4 |
| Grade 3–4 TRAEs, n (%) | - | 26 (31.3) | - | 12 (24.0) |  | - | 32 (28.3) | - | 4 (21.1) |
| TRAEs leading to discontinuation, n (%) |  |  |  |  |  |  |  |  |  |
| All components of the regimen | 21 (25.3) | 13 (15.7) | 12 (24.0) | 8 (16.0) |  | 29 (25.7) | 18 (15.9) | 5 (26.3) | 3 (15.8) |
| Any components of the regimen | 28 (33.7) | 17 (20.5) | 14 (28.0) | 9 (18.0) |  | 37 (32.7) | 22 (19.5) | 5 (26.3) | 3 (15.8) |
| Treatment-related deaths, n (%) | 3 (3.6) | | 1 (2.0) | |  | 3 (2.7) | | 2 (10.5) | |

The severity of adverse events was assessed according to CTCAE v 5.0.

^a^ Data were unknown for 8 patients.

^b^ Data were unknown for 9 patients.

CTCAE, Common Terminology Criteria for Adverse Events Grading System; ECOG PS, Eastern Cooperative Oncology Group performance status; TRAE, treatment-related adverse event.

Supplementary Table 7. Associations of clinical factors with grade ≥ 3 irAE in overall population

|  |  |  | Grade ≥ 3 irAE, n (%) |  |  | Univariate analysis |  |  | Multivariate analysis |  |
| --- | --- | --- | --- | --- | --- | --- | --- | --- | --- | --- |
|  |  | *N* | Without | With |  | Odds ratio (95% CI) | P-value |  | Odds ratio (95% CI) | P-value |
| Age | < 75 years | 263 | 178 (67.7) | 85 (32.3) |  |  | 0.564 |  |  |  |
|  | ≥ 75 years | 73 | 52 (71.2) | 21 (28.8) |  | 0.85 (0.48–1.49) |  |  |  |  |
| Sex | Female | 71 | 47 (66.2) | 24 (33.8) |  |  | 0.674 |  |  |  |
|  | Male | 282 | 194 (68.8) | 88 (31.2) |  | 0.89 (0.51–1.54) |  |  |  |  |
| ECOG PS | 0–1 | 313 | 212 (67.7) | 101 (32.3) |  |  | 0.799 |  |  |  |
|  | ≥ 2 | 30 | 21 (70.0) | 9 (30.0) |  | 0.90 (0.40–2.03) |  |  |  |  |
| Smoking status | Never | 30 | 22 (73.3) | 8 (26.7) |  |  | 0.535 |  |  |  |
|  | Former or current | 323 | 219 (67.8) | 104 (32.2) |  | 1.31 (0.56–3.03) |  |  |  |  |
| Histology | Adenocarcinoma | 222 | 153 (68.9) | 69 (31.1) |  |  | 0.340 |  |  |  |
|  | Squamous | 93 | 59 (63.4) | 34 (36.6) |  | 1.28 (0.77–2.13) |  |  |  |  |
|  | Other | 38 | 29 (76.3) | 9 (23.7) |  | 0.69 (0.31–1.53) |  |  |  |  |
| Clinical stage | IV | 276 | 190 (68.8) | 86 (31.2) |  |  | 0.664 |  |  |  |
|  | Recurrence | 77 | 51 (66.2) | 26 (33.8) |  | 1.13 (0.66–1.93) |  |  |  |  |
| Previous radiation | No | 246 | 161 (65.4) | 85 (34.6) |  |  | 0.085 |  |  | 0.283 |
|  | Yes | 107 | 80 (74.8) | 27 (25.2) |  | 0.64 (0.38–1.06) |  |  | 0.75 (0.44–1.27) |  |
| Metastasis | No | 57 | 45 (78.9) | 12 (21.1) |  |  | 0.062 |  |  | 0.073 |
|  | Yes | 296 | 196 (66.2) | 100 (33.8) |  | 1.91 (0.97–3.78) |  |  | 1.88 (0.94–3.73) |  |
| Tumor PD-L1 expression | < 1% | 163 | 114 (69.9) | 49 (30.1) |  |  | 0.470 |  |  |  |
|  | 1–49% | 123 | 82 (66.7) | 41 (33.3) |  | 1.16 (0.70–1.92) |  |  |  |  |
|  | ≥ 50% | 29 | 17 (58.6) | 12 (41.4) |  | 1.64 (0.73–3.70) |  |  |  |  |
| Interstitial lung disease | No | 346 | 236 (68.2) | 110 (31.8) |  |  | 0.856 |  |  |  |
|  | Past or current | 7 | 5 (71.4) | 2 (28.6) |  | 0.86 (0.16–4.49) |  |  |  |  |
| Autoimmune disease | No | 345 | 236 (68.4) | 109 (31.6) |  |  | 0.723 |  |  |  |
|  | Yes | 8 | 5 (62.5) | 3 (37.5) |  | 1.30 (0.30–5.53) |  |  |  |  |
| CRP | Normal | 75 | 50 (66.7) | 25 (33.3) |  |  | 0.658 |  |  |  |
|  | Abnormal | 274 | 190 (69.3) | 84 (30.7) |  | 0.88 (0.51–1.52) |  |  |  |  |
| LDH | Normal | 192 | 135 (70.3) | 57 (29.7) |  |  | 0.348 |  |  |  |
|  | Abnormal | 154 | 101 (65.6) | 53 (34.4) |  | 1.24 (0.79–1.96) |  |  |  |  |
| NLR | Abnormal | 319 | 219 (68.7) | 100 (31.3) |  |  | 0.639 |  |  |  |
|  | Normal | 34 | 22 (64.7) | 12 (35.3) |  | 1.19 (0.57–2.51) |  |  |  |  |
| WBC | Normal | 256 | 177 (69.1) | 79 (30.9) |  |  | 0.569 |  |  |  |
|  | Abnormal | 97 | 64 (66.0) | 33 (34.0) |  | 1.16 (0.70–1.90) |  |  |  |  |
| Lymphocyte | Normal | 261 | 170 (65.1) | 91 (34.9) |  |  | 0.034 |  |  | 0.074 |
|  | Abnormal | 92 | 71 (77.2) | 21 (22.8) |  | 0.55 (0.32–0.96) |  |  | 0.59 (0.34–1.05) |  |
| Neutrophil | Normal | 349 | 237 (67.9) | 112 (32.1) |  |  | 0.984 |  |  |  |
|  | Abnormal | 4 | 4 (100) | 0 (0) |  | - |  |  |  |  |

The severity of adverse events was assessed according to CTCAE v 5.0.

CRP, normal: 0.00–0.14 mg/dL; LDH, normal: 124–222 U/L; NLR, normal: < 2.0; WBC, normal: 3.3–8.6 × 10^3^/μL; lymphocyte, normal: ≥ 1.0 x10^3^/μL; neutrophil, normal: ≥ 2.0 × 10^3^/μL.

CI, confidence interval; CRP, c-reactive protein; CTCAE, Common Terminology Criteria for Adverse Events Grading System; ECOG PS, Eastern Cooperative Oncology Group performance status; irAE, immune-related adverse event; LDH, lactate dehydrogenase; NLR, neutrophil-to-lymphocyte ratio; PD-L1, programmed cell death ligand 1 WBC, white blood cell.

Supplementary Table 8. Objective response by subgroups in patients with nivolumab plus ipilimumab with chemotherapy

|  | Age | |  | Histology | | |  | ECOG PS | |  | Tumor PD-L1 expression | | |
| --- | --- | --- | --- | --- | --- | --- | --- | --- | --- | --- | --- | --- | --- |
|  | < 75 years | ≥ 75 years |  | Adenocarcinoma | Squamous | Other |  | 0–1 | ≥ 2 |  | < 1% | 1–49% | ≥ 50% |
|  | *N* = 130 | *N* = 12 |  | *N* = 110 | *N* = 29 | *N* = 10 |  | *N* = 144 | *N* = 5 |  | *N* = 72 | *N* = 46 | *N* = 14 |
| Objective responses, *n* (%) | 49 (37.7) | 6 (50.0) |  | 43 (39.1) | 12 (41.4) | 4 (40.0) |  | 57 (39.6) | 2 (40.0) |  | 26 (36.1) | 21 (45.7) | 10 (71.4) |
| (95% CI) | (29.3–46.6) | (21.1–78.9) |  | (29.9–48.9) | (23.5–61.1) | (12.2–73.8) |  | (31.5–48.1) | (5.3–85.3) |  | (25.1–48.3) | (30.9–61.0) | (41.9–91.6) |
| Best overall response, *n* (%) |  |  |  |  |  |  |  |  |  |  |  |  |  |
| Complete response | 1 (0.8) | 0 (0) |  | 0 (0) | 1 (3.4) | 0 (0) |  | 1 (0.7) | 0 (0) |  | 1 (1.4) | 0 (0) | 0 (0) |
| Partial response | 48 (36.9) | 6 (50.0) |  | 43 (39.1) | 11 (37.9) | 4 (40.0) |  | 56 (38.9) | 2 (40.0) |  | 25 (34.7) | 21 (45.7) | 10 (71.4) |
| Stable disease | 29 (22.3) | 1 (8.3) |  | 24 (21.8) | 3 (10.3) | 4 (40.0) |  | 31 (21.5) | 0 (0) |  | 17 (23.6) | 10 (21.7) | 1 (7.1) |
| Progressive disease | 45 (34.6) | 5 (41.7) |  | 39 (35.5) | 11 (37.9) | 2 (20.0) |  | 50 (34.7) | 2 (40.0) |  | 27 (37.5) | 12 (26.1) | 1 (7.1) |
| Not evaluable | 7 (5.4) | 0 (0) |  | 4 (3.6) | 3 (10.3) | 0 (0) |  | 6 (4.2) | 1 (20.0) |  | 2 (2.8) | 3 (6.5) | 2 (14.3) |
| Disease controls, *n* (%) | 78 (60.0) | 7 (58.3) |  | 67 (60.9) | 15 (51.7) | 8 (80.0) |  | 88 (61.1) | 2 (40.0) |  | 43 (59.7) | 31 (67.4) | 11 (78.6) |
| (95% CI) | (51.0–68.5) | (27.7–84.8) |  | (51.1–70.1) | (32.5–70.6) | (44.4–97.5) |  | (52.6–69.1) | (5.3–85.3) |  | (47.5–71.1) | (52.0–80.5) | (49.2–95.3) |
| Median DoR, months (95% CI) | 6.0 (4.2–NE) | NR (0.7–NE) |  | NR (4.2–NE) | 4.1 (3.0–NE) | NR (NE–NE) |  | 6.1 (4.2–NE) | 4.2 (NE–NE) |  | 6.1 (4.1–NE) | 4.2 (3.0–NE) | 5.2 (4.2–NE) |
| Median PFS, months (95% CI) | 5.7 (4.9–7.4) | NR (5.1–NE) |  | 6.4 (4.7–7.6) | 5.3 (4.9–7.7) | 10.2 (NE–NE) |  | 6.7 (5.3–7.7) | 2.2 (1.6–5.3) |  | 5.9 (4.4–NE) | 6.7 (5.0–10.2) | 6.4 (5.3–NE) |

The number of patients was the number for whom the results of the best overall response were available. CI, confidence interval; DoR, duration of response; ECOG PS, Eastern Cooperative Oncology Group performance status; NE, not evaluable; NR, not reached; PD-L1, programmed cell death ligand 1; PFS, progression-free survival.

Supplementary Table 9. Objective response by subgroups in patients with nivolumab plus ipilimumab

|  | Age | |  | Histology | | |  | ECOG PS | |  | Tumor PD-L1 expression | | |
| --- | --- | --- | --- | --- | --- | --- | --- | --- | --- | --- | --- | --- | --- |
|  | < 75 years | ≥ 75 years |  | Adenocarcinoma | Squamous | Other |  | 0–1 | ≥ 2 |  | < 1% | 1–49% | ≥ 50% |
|  | *N* = 55 | *N* = 31 |  | *N* = 47 | *N* = 34 | *N* = 11 |  | *N* = 78 | *N* = 11 |  | *N* = 42 | *N* = 42 | *N* = 4 |
| Objective responses, *n* (%) | 23 (41.8) | 11 (35.5) |  | 15 (31.9) | 16 (47.1) | 3 (27.3) |  | 28 (35.9) | 5 (45.5) |  | 13 (31.0) | 16 (38.1) | 2 (50.0) |
| (95% CI) | (28.7–55.9) | (19.2–54.6) |  | (19.1–47.1) | (29.8–64.9) | (6.0–61.0) |  | (25.3–47.6) | (16.7–76.6) |  | (17.6–47.1) | (23.6–54.4) | (6.8–93.2) |
| Best overall response, *n* (%) |  |  |  |  |  |  |  |  |  |  |  |  |  |
| Complete response | 0 (0) | 2 (6.5) |  | 1 (2.1) | 1 (2.9) | 0 (0) |  | 1 (1.3) | 1 (9.1) |  | 2 (4.8) | 0 (0) | 0 (0) |
| Partial response | 23 (41.8) | 9 (29.0) |  | 14 (29.8) | 15 (44.1) | 3 (27.3) |  | 27 (34.6) | 4 (36.4) |  | 11 (26.2) | 16 (38.1) | 2 (50.0) |
| Stable disease | 15 (27.3) | 8 (25.8) |  | 11 (23.4) | 9 (26.5) | 4 (36.4) |  | 23 (29.5) | 1 (9.1) |  | 8 (19.0) | 15 (35.7) | 0 (0) |
| Progressive disease | 16 (29.1) | 11 (35.5) |  | 20 (42.6) | 8 (23.5) | 4 (36.4) |  | 27 (34.6) | 3 (27.3) |  | 19 (45.2) | 11 (26.2) | 2 (50.0) |
| Not evaluable | 1 (1.8) | 1 (3.2) |  | 1 (2.1) | 1 (2.9) | 0 (0) |  | 0 (0) | 2 (18.2) |  | 2 (4.8) | 0 (0) | 0 (0) |
| Disease controls, *n* (%) | 38 (69.1) | 19 (61.3) |  | 26 (55.3) | 25 (73.5) | 7 (63.6) |  | 51 (65.4) | 6 (54.5) |  | 21 (50.0) | 31 (73.8) | 2 (50.0) |
| (95% CI) | (55.2–80.9) | (42.2–78.2) |  | (40.1–69.8) | (55.6–87.1) | (30.8–89.1) |  | (53.8–75.8) | (23.4–83.3) |  | (34.2–65.8) | (58.0–86.1) | (6.8–93.2) |
| Median DoR, months (95% CI) | 6.6 (3.5–6.8) | NR (4.4–NE) |  | 6.8 (6.6–NE) | NR (3.0–NE) | 4.9 (NE–NE) |  | 6.6 (4.9–NE) | NR (NE–NE) |  | NR (4.9–NE) | NR (3.0–NE) | 6.7 (6.6–6.8) |
| Median PFS, months (95% CI) | 6.1 (4.1–7.2) | 5.9 (3.8–NE) |  | 6.1 (2.8–9.3) | 5.9 (4.6–NE) | 4.7 (2.7–NE) |  | 6.3 (5.3–9.1) | 1.4 (0.8–4.8) |  | 4.2 (2.8–7.0) | 6.3 (4.7–NE) | 6.8 (3.0–10.8) |

The number of patients was the number for whom the results of the best overall response were available. CI, confidence interval; DoR, duration of response; ECOG PS, Eastern Cooperative Oncology Group performance status; NE, not evaluable; NR, not reached; PD-L1, programmed cell death ligand 1; PFS, progression-free survival.
